# Temporal Patterns of Nucleotide Misincorporations and DNA Fragmentation in Ancient DNA

**DOI:** 10.1371/journal.pone.0034131

**Published:** 2012-03-30

**Authors:** Susanna Sawyer, Johannes Krause, Katerina Guschanski, Vincent Savolainen, Svante Pääbo

**Affiliations:** 1 Max Planck Institute for Evolutionary Anthropology, Leipzig, Germany; 2 Institute for Archaeological Sciences, University of Tübingen, Tübingen, Germany; 3 Center for Integrative Genomics, University of Lausanne, Lausanne, Switzerland; 4 Silwood Park Campus, Imperial College London, Ascot, Berkshire, United Kingdom; Institut de Biologia Evolutiva - Universitat Pompeu Fabra, Spain

## Abstract

DNA that survives in museum specimens, bones and other tissues recovered by archaeologists is invariably fragmented and chemically modified. The extent to which such modifications accumulate over time is largely unknown but could potentially be used to differentiate between endogenous old DNA and present-day DNA contaminating specimens and experiments. Here we examine mitochondrial DNA sequences from tissue remains that vary in age between 18 and 60,000 years with respect to three molecular features: fragment length, base composition at strand breaks, and apparent C to T substitutions. We find that fragment length does not decrease consistently over time and that strand breaks occur preferentially before purine residues by what may be at least two different molecular mechanisms that are not yet understood. In contrast, the frequency of apparent C to T substitutions towards the 5′-ends of molecules tends to increase over time. These nucleotide misincorporations are thus a useful tool to distinguish recent from ancient DNA sources in specimens that have not been subjected to unusual or harsh treatments.

## Introduction

When endogenous DNA is preserved in ancient tissues found in settings other than the permafrost, it generally occurs in only small amounts [1] and in the presence of much larger amounts of DNA from microbes that colonized specimens after the death of the organisms [2], [3]. The DNA extracted from such remains is invariably modified in various ways. In particular, three features of ancient DNA have been described [4]–[6]: (*i*) short fragment length [6]; (*ii*) an increased occurrence of purines (adenine (A) and guanosine (G) residues) before strand breaks [4], putatively due to depurination of the DNA followed by hydrolysis of the phosphate-sugar backbone; (*iii*) an increased frequency of apparent cytosine (C) to thymine (T) substitutions close to the ends of the fragments [4], [5], presumably due to deamination of cytosine residues that occur primarily in the single-stranded overhangs of DNA fragments [4], [5].

Knowledge of how these features of ancient DNA accumulate over time is of interest in itself [7] and may also allow DNA of different ages to be identified and distinguished from each other [8]. This is practically important since ancient DNA experiments are highly susceptible to contamination by modern DNA [1], [9], [10]. Although various measures can minimize the risk that contaminating DNA is introduced during laboratory and excavation procedures [4], [11], modern DNA that is present in a specimen as it arrives in the laboratory cannot be avoided. This is particularly relevant when remains of anatomically modern humans are investigated as human DNA frequently contaminates ancient bones [1], [12] and cannot easily be distinguished by its sequences from DNA of ancient human remains. Therefore, some studies of ancient modern humans use the fact that their samples come from populations carrying DNA sequence variants that are rare in present-day populations as an argument for authenticity [13], [14]. In other studies DNA sequences have been determined from all humans believed to have come into contact with a specimen in order to exclude contamination [15]. However, such measures cannot exclude all possible sources of contamination and they limit the projects that can be undertaken. It would therefore be very useful if molecular features endogenous to the ancient DNA could be used to support the claim that a population of DNA molecules is of old age. The three features of ancient DNA above have been used in the study of a 32,000-year-old early modern human from Russia [8]. However, the more general application of this approach is hampered by the fact that it is largely unknown if, and if so how rapidly, these features accumulate over time. Here, we have analyzed DNA extracted from 86 animal and Neandertal samples that vary in age from 18 years to ∼60,000 years and differ with respect to the environments in which they have been preserved.

## Results

DNA was extracted from remains of 44 primates, 31 horses and 5 cows ranging in age from 18 to 2,400 years old. The primates were collected in Africa over the last 100 years and have since been stored in museums. Nine of the gorillas had been roasted over fire and buried in the ground for a few months before being collected. At the Museum für Naturkunde, Berlin (MfN) they were treated with “ponal glue” (a polyvinyl acetate based wood glue). Four of the 13 monkeys from the same museum have been treated with “Leipzig cocktail” (a sulfuric acid based preservative) (Table S1). For the other primates no information about treatment in the museum is available. The horse and cow bones were excavated at open-air sites in Holland and Germany, cleaned by hand or brush, and then stored in museums for between 5 and 60 years (Table S1).

Aliquots of the DNA extracts were used to construct DNA sequencing libraries [16] that were then amplified by the polymerase chain reaction (PCR) using primers specific to the library adaptors and a non-proof reading DNA polymerase. DNA fragments from the mitochondrial (mt) genomes were captured [17] and sequenced on the Illumina GAII platform using paired-end 75- or 101-bp reads. Paired reads were merged and the resultant DNA sequences aligned to the mtDNAs from the relevant species. The fraction of DNA sequences that mapped to the mitochondrial genomes varied from 0.02% (three monkeys) to 52% (a 48-year-old-monkey) (Table S1). DNA sequences with identical start and end coordinates in the mtDNA were collapsed into unique sequences. The number of such unique sequences varied from 20 (a 104-year-old monkey) to 345,619 (an 83-year-old-gorilla) (Table S1). In addition, mtDNA data from six Neandertal sequencing libraries with 0.2–2.9% modern human contamination [4], [18] were included in the analyses.

**Figure 1:**
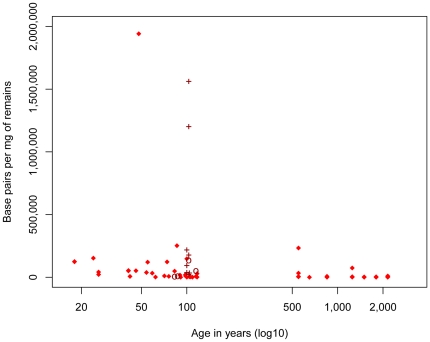
Amounts of endogenous mtDNA sequences (bp) determined per milligram (mg) of tissue as a function of age. Note that since the Neandertal specimens were all ascertained for containing endogenous DNA they are excluded from this analysis. Nine samples known to have been “roasted” over fire and treated with ponal glue are indicated by crosses and four samples treated by the “Leipzig cocktail” are indicated by circles.

We first estimated the amount of endogenous mtDNA preserved in the specimens by calculating the number of base pairs (bp) sequenced per milligram (mg) of tissue for all 80 animal samples analyzed. The amount of DNA varied from 12 bp/mg (a 600–700-year-old horse) to 1,941,450 bp/mg (50-year-old monkey). Despite a very large variation in DNA amounts, there is a significant negative correlation between amounts of endogenous DNA and age (*rho* = −0.625, p-value = 5.90e-10) (Fig. 1). The removal of three young samples which contain almost an order of magnitude more DNA than the others only slightly reduces the correlation (*rho* = −0.623, p-value = 1.44e-09) (Fig. 1).

**Figure 2:**
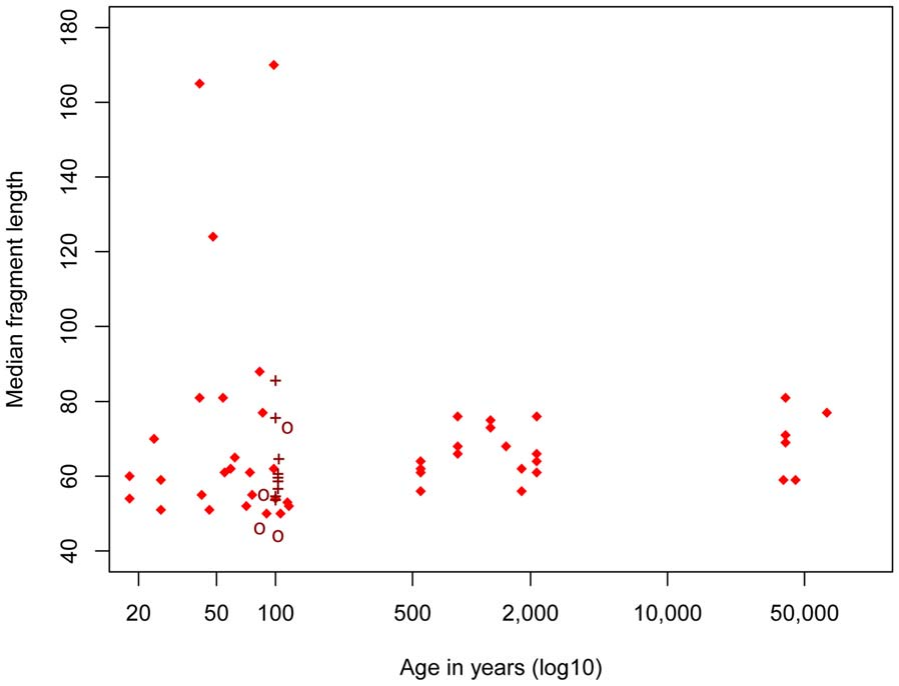
Median length of endogenous mtDNA fragment as a function of age. Nine samples known to have been “roasted” over fire and treated with ponal glue are indicated by crosses and four samples treated by the “Leipzig cocktail” are indicated by circles.

Analyses of patterns of DNA degradation and damage were restricted to samples that yielded more than 450 mtDNA unique sequences. This was the case for 39 of the 44 primates and 20 of the 36 horse and cow samples (Table S1). The median lengths of the mtDNA sequences from the 65 animal and Neandertal libraries vary from 44 to 170 bp (Fig. 2). The three samples with sizes above 100 bp are all less than 100 years old (Table S1). However, samples of similar young age have fragment sizes as low as 44 bp and thus overlap with samples that are up to 60,000 years old (Table S2, Fig. 2). Thus, although the samples range in age from 18 years to approximately 60,000 years, there is no significant correlation between median fragment sizes and age (*rho* = 0.19, p-value = 0.15). Removing the primate samples known to have been heated and treated with chemicals from the analysis does not result in a significant correlation (*rho* = 0.20, p-value = 0.12).

**Figure 3:**
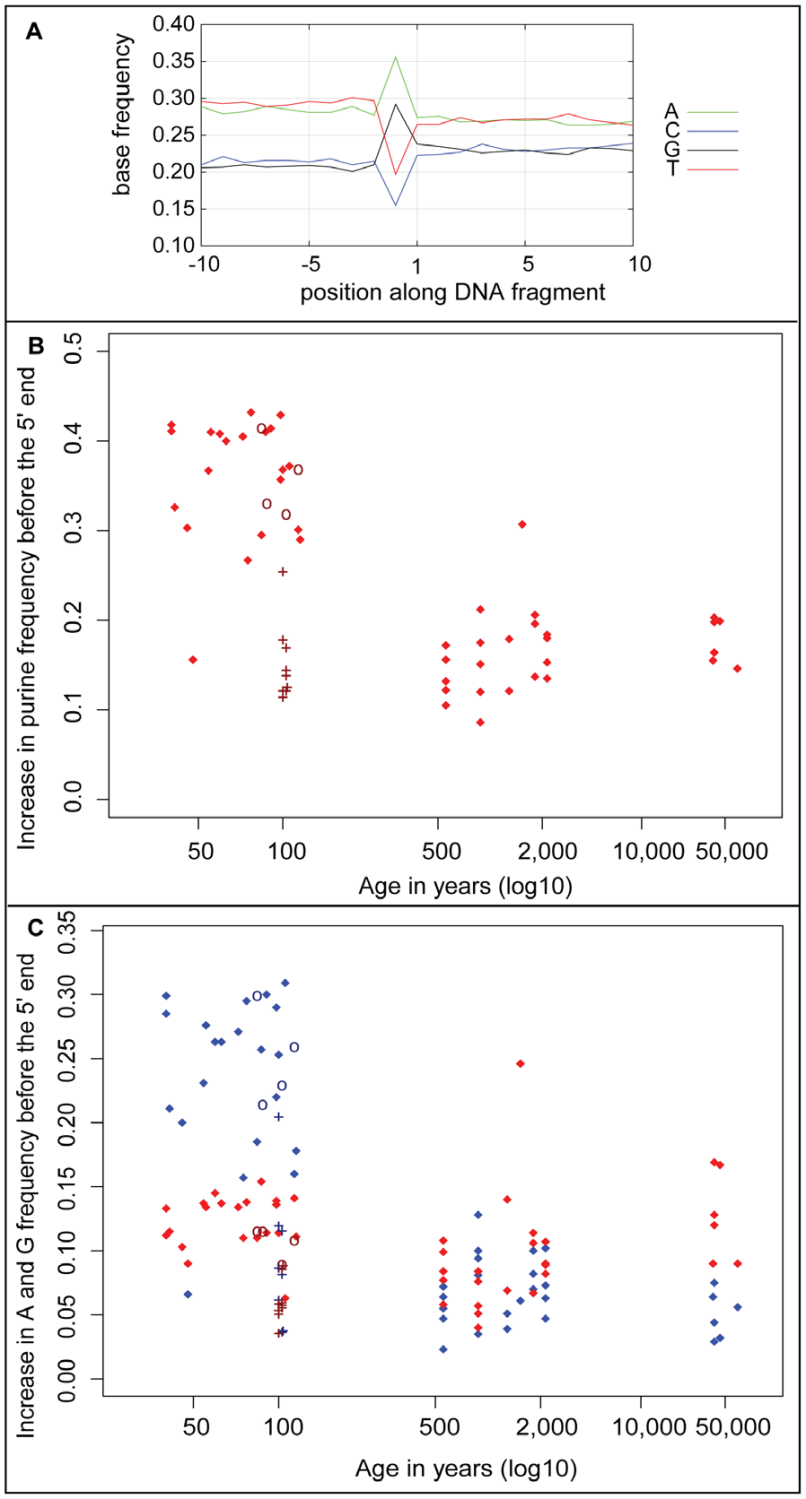
Characteristics of purine frequency prior to strand breaks. **A:** Base frequencies 5′ and 3′ of 5′-ends of endogenous mtDNA fragments of a horse (sample 54). **B:** Increase in purine frequency at position immediately 5′ (position −1) of mtDNA fragment ends relative to positions −5 to −10 as a function of age. Nine samples known to have been “roasted” over fire and treated with ponal glue are indicated by crosses and four samples treated by the “Leipzig cocktail” are indicated by circles. **C:** Increase in A (blue) and G (red) frequencies at position −1 of mtDNA fragment. Nine samples known to have been “roasted” over fire and treated with ponal glue are indicated by crosses and four samples treated by the “Leipzig cocktail” are indicated by circles.

In order to investigate if particular nucleotide residues tend to occur adjacent to DNA strand breaks, each ancient DNA fragment was aligned to its corresponding reference sequence. While 5′-ends in the sequencing libraries represent actual 5′-ends in the ancient DNA, overhanging 3′-ends are removed by the exonucleolytic activity of *T4* DNA polymerase during library preparation while recessed 3′-ends are filled in by the same enzyme. Thus, 3′-ends in the molecules sequenced represent the 5′-ends of the complementary DNA strands in the ancient DNA molecules. We recorded the frequencies of the four nucleotides in the reference sequences adjacent to the 5′-ends of the DNA fragments. As previously described [4], we find that the purines A and G are overrepresented adjacent to 5′-ends of the ancient DNA fragments (Fig. 3A). To gauge this excess of purines we subtracted the average purine frequency between positions 5 to 10 before the 5′-ends from the purine frequencies at the position immediately preceding 5′-ends of fragments. These differences vary from 0.09 to 0.43 (Fig. 3B). Interestingly, many samples that are about 100 years or younger have purine overrepresentations of 0.2 and higher while most samples older than 500 years have overrepresentations of 0.2 and lower. Thus, there is a negative correlation between purine frequency immediately adjacent to the 5′-ends of ancient DNA fragments and age of the samples (*rho* = −0.57, p-value = 5.1e-07). Eight of the nine “ponal glue”-treated gorillas show purine overrepresentations between 0.11 and 0.18 and the four “Leipzig cocktail”-treated monkeys have purine overrepresentations of 0.33 and 0.41 (Tables S1 and S2). Removal of these samples results in a slightly stronger negative correlation (*rho* = −0.63, p-value = 5.0e-07). Interestingly, samples 100 years or younger, tend to show a greater A than G overrepresentation, especially when the samples that were heated and treated with ponal glue are disregarded (Fig. 3C). Conversely, the samples older than 40,000 years all have a greater G than A overrepresentation.

**Figure 4:**
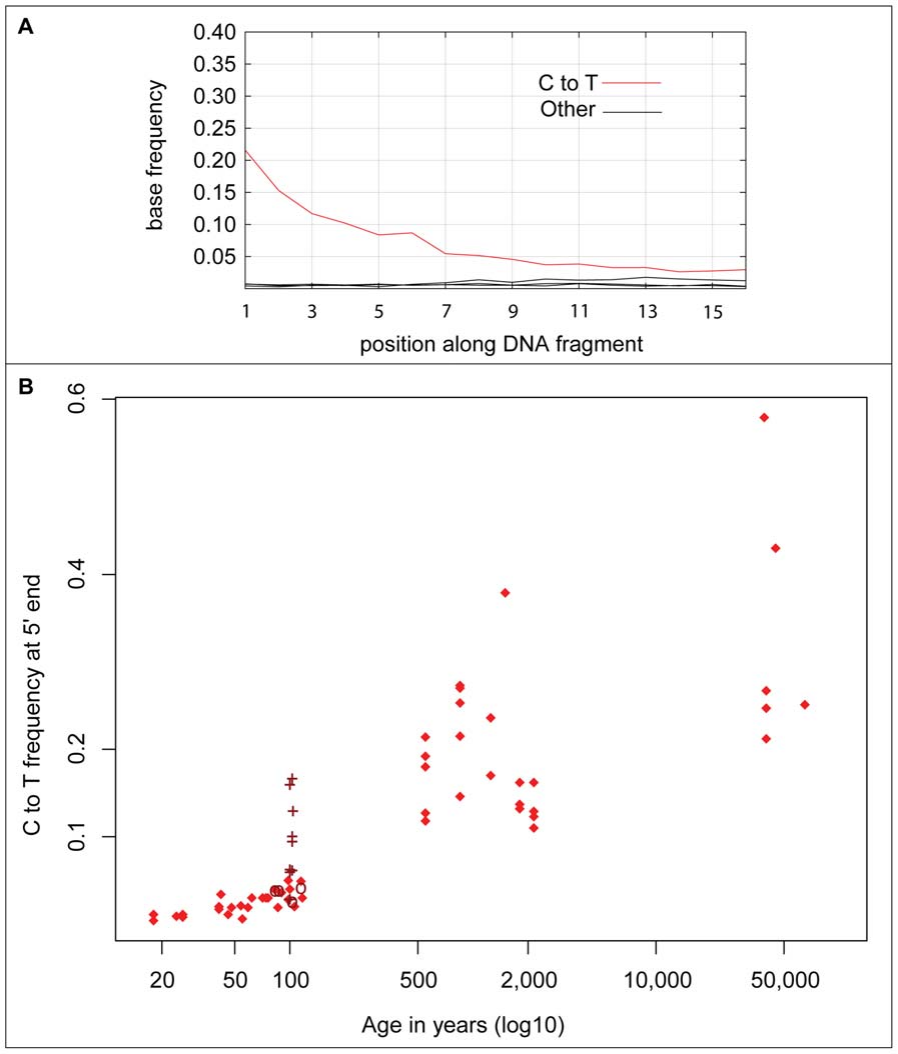
Characteristics of C to T misincorporations. **A:** C to T misincorporations at the first 15 bases of endogenous mtDNA fragments from of a 500–600 year old horse sample (sample 54). **B:** C to T misincorporations at the first position of mtDNA fragments as a function of age. Nine samples known to have been “roasted” over fire and treated with ponal glue are indicated by crosses and four samples treated by the “Leipzig cocktail” are indicated by circles.

Patterns of nucleotide misincorporations were analyzed by comparison of the mtDNA fragments to their corresponding reference sequences (see Materials and Methods for reference sequences) and determination of the frequency of apparent substitutions at each position along the sequences. As previously described [4], [5], the frequency of apparent C to T substitutions increases towards 5′-ends of the fragments (see Fig. 4A for an example). Due to the treatment of the ancient DNA with *T4* DNA polymerase prior to adaptor ligation, apparent G to A substitutions towards the 3′-ends reflect miscoding lesions towards the 5′-ends of the complimentary DNA strands [4]. We restricted these analyses to the 5′-ends of the DNA sequences. Over-all, there is a strong positive correlation between such apparent C to T substitutions and age (*rho* = 0.87, p-value<2.2e-16) (Fig. 4B). It is notable that eight of the nine 100-year-old gorilla specimens that have been heated over fire and treated with “ponal glue” have C to T frequencies between 0.06 and 0.16 and are thus higher than the other samples in the same age range. Disregarding these samples, the highest C to T frequency of the non-treated samples that are 117 years and younger is 0.05, while the lowest C to T frequency for samples 500 years and older is 0.11 (Table S2).

## Discussion

The depositional conditions to which archaeological specimens have been exposed are often largely unknown. This is unfortunate since many aspects of these conditions are of relevance for DNA preservation. They include - but are not limited to - temperature, acidity, amount of water percolation, salinity, and presence of minerals to which nucleic acids may be adsorbed. This lack of information often applies also to how specimens have been stored in museum collections where environmental conditions may not be well controlled and cleaning and treatment of the specimens with various preservatives are often not documented. Given that preservation conditions are thus variable and largely unknown it may seem unlikely that any particular aspect of DNA preservation would correlate with age of samples.

In an attempt to nevertheless identify molecular features of DNA preservation where DNA endogenous to an ancient specimen differs from recent DNA that contaminates it, we have previously analyzed mtDNA sequences isolated from three Neandertal bones that were heavily contaminated with modern human DNA. It was found that whereas DNA fragment sizes often overlap between the Neandertal mtDNA and contaminating modern human DNA, in two cases the latter tended to be longer. When the base composition close to 5′-ends of the mtDNA sequences was analyzed, the endogenous Neandertal mtDNA showed an elevation of G and A residues at the positions immediately 5′ to fragment ends whereas the modern human contaminants did not. Finally, in agreement with the observation that cytosine deamination, a common modification in ancient DNA that cause nucleotide misincorporations [4], tends to accumulate at the ends of molecules [4], it was found that cytosine residues at the 5′-ends of endogenous Neandertal mtDNA molecules appeared as thymine residues in around a third of the cases, whereas this was the case in less than 5% in the contaminating mtDNA molecules. A combination of these three features was then used to argue that the mtDNA sequence determined from a 32,000-year-old modern human from the Kostenki site in Russia were endogenous to the specimen [8].

Here, we find that fragment size does not decrease in a consistent manner over time. In fact, with few exceptions, endogenous mtDNA prepared from samples that are less than 100 years old are below 100 bp in length and do not differ in size from mtDNAs from much older specimens (Fig. 2). Thus, as has been previously suggested [6], in most cases degradation of the DNA to a small average size happens rapidly after death, probably as a result of autolytic processes. However, although the average size of fragments retrieved from older specimens can be larger than those retrieved from younger specimens, over-all the total amounts of mtDNA retrievable from specimens decrease over time, in particular due to the fact that the fraction of specimens that do not contain any detectable endogenous DNA increases (Fig. 1).

Interestingly, some of the mechanisms by which strand breaks occur may differ in older and younger specimens. This is suggested by the fact that purine residues occur immediately 5′ of strand breaks more often in the younger specimens (Fig. 3B). It is also suggested by the fact that while A residues predominate over G residues in most samples younger than 100 years, the two purines occur approximately equally often in samples 500–2,000 years old while G residues predominate in the Neandertal samples which are over 40,000 years old (Fig. 3C). Since the rate of hydrolytic loss of guanine from DNA is slightly more rapid than adenine loss [7], this suggests that a chemical mechanism other than hydrolytic depurination may underlie the strand breaks that occur soon after death and that this mechanism has a propensity to cause strand breaks 3′to A residues. Plausible candidates may be autolytic enzymes. For example, many lysosomal nucleases tend to cut nucleic acid chains on the 3′-side of their recognition sites and often prefer A–T base pairs (T. Lindahl, personal communication). The slower mechanism, which creates breaks preferentially at G residues, may be more enigmatic, but could include oxidative processes.

Nucleotide misincorporations at the 5′-ends of DNA fragments differ from the other two features in that they exhibit a strong and positive correlation with age. This may seem surprising given that the rate of hydrolytic deamination of cytosine residues depends on factors such as temperature, pH, and salinity which may vary depending on depositional conditions. Indeed, that such factors do play a role is suggested by the fact that eight of the nine gorilla samples that have been heated over fire have C to T frequencies that are higher than the other samples of similar age. Nevertheless, in samples younger than 100 years less than 20% of C residues at the 5′-ends of DNA sequences appear as T residues whereas in all Neandertal samples more than 20% of such Cs appear as Ts. If the “roasted” gorilla samples are disregarded, less than 10% of Cs at the first positions of sequences appear as Ts in all samples younger than 100 years while more than 10% do so in all samples older than 500 years.

The C to T nucleotide misincorporation pattern close to the ends of DNA fragments thus seems to offer itself as a means to support the claim that a population of DNA molecules is of old age. We suggest that in conjunction with experimental controls and other analyses, in particular deep sequencing to show that the DNA comes exclusively or predominantly from a single individual [8], this can be used to support the claim that DNA sequences determined from samples that have not been heated or otherwise exposed to unusual conditions are old. It should be noted though that some procedures used to remove contamination from the surface of ancient specimens, in particular treatment with bleach, may increase the rate of deamination in contaminating DNA that survive the treatment [19]. It is hoped that when other molecular features of DNA extracted from organic remains, for example how strand breaks occur at adenine and guanines residues, are better understood they will also be useful for gauging the age of populations of DNA sequences.

## Materials and Methods

### DNA extraction

All samples were handled in a clean room facility designed to avoid contamination. Bones were drilled with a dentist drill at 10,000 rpm to produce up to 400 mg of bone powder. Other tissues were ground with a mortar and pestle. DNA was extracted from tissue powder using EDTA and proteinase K followed by binding to silica [20] to produce 100 uL of DNA extract.

### Library preparation and amplification

DNA sequencing libraries were made from 20–30 uL of the DNA extracts using Illumina Multiplex adaptors [16], except for sample 106 and the gorilla samples. For these 11 extracts, the Illumina Multiplex adapters had a special four base pair key on each end of the insert (5′AATGATACGGCGACCACCGAGATCTACACTCTTTCCCTACACGACGCTCTTCCGATCT**ACTC** – insert – **GAGT**AGATCGGAAGAGCACACGTCTGAACTCCAGTCACIIIIIIIATCTCGTATGCCGTCTTCTGCTTG 3′, the key sequence is in bold). To create the libraries, ends of DNA molecules are made blunt by *T4* DNA polymerase, which removes 3′ overhanging ends and fills in 3′ recessed ends. The latter will result in the addition of nucleotides that carry complementary and reversed versions of the 5′-ends of the opposite strands. Quantitative PCR (qPCR) was used to determine the amounts of DNA in the libraries before and after every amplification and capture.

After library preparation, adaptors carrying DNA sequence indices specific for each sample were added by PCR [16], using 45 uL of water, 6 uL of 10× Thermapol Buffer (NEB), 1 uL of 25 mM dNTPs (Fermentas), 4 uL of 10 uM IS4_indPCR.P5 primer [16], 4 uL of 10 uM Indexing primer, 1 uL of 5 U/uL AmpliTaq Gold (Applied Biosystems) and 38 uL of template with the following thermal profile: initialization for 12 minutes at 95°C, 10 cycles of denaturation for 20 seconds at 95°C, annealing for 30 seconds at 60°C, elongation for 40 seconds at 72°C, and a final elongation of 5 minutes at 72°C.

All subsequent PCRs were done using 50 uL of 2× Phusion Master Mix (Fermentas), 4 uL of 10 uM IS5_reamp.P5/IS6_ reamp.P7 [16] each, 37 uL of water and 5 uL of template and initialization for 30 seconds at 98°C, denaturation for 20 seconds at 98°C, annealing for 30 seconds at 60°C, elongation for 40 seconds at 72°C, and a final elongation of 5 minutes at 72°C. The number of cycles was calculated to avoid saturation using the qPCR result [16].

### Mitochondrial enrichment and sequencing

With the exception of the gorillas which were analyzed individually, all amplified libraries were pooled by species and enriched for mitochondrial DNA using the targeted region capture on streptavidine coated magnetic beads [17]. The closest possible species was used as bait for capture (Table S1). All capture pools were sequenced directly with no or a maximum of 6 cycles (horse and cow pools) of amplification. The library pools were sequenced in six lanes of 5 Illumina flow cells (Cluster Generation kit V4, sequencing chemistry V4) on the Illumina GAIIx platform. The manufacturer's instructions were followed.

The run was processed with SCS/RTA 1.6 (Illumina Inc.) and the PhiX 174 control reads, which were spiked into each lane or run on a dedicated lane, were used as a training data set for the base caller Ibis [21]. Raw reads were separated by their index reads (requiring perfect matches with the indices; [16]) and the two reads from each cluster merged by requiring at least an 11nt overlap [22]. In the overlapping read parts, the base with the highest base quality score was called. Fused sequences having more than 5 bases with a quality score [23] below 15 were removed. For sample 106 and the gorillas, which had a special key sequence in the adapters, we required that the key sequences had an exact match, after which the key sequences were trimmed off.

The merged sequences were aligned to the closest available reference mtDNAs (*Gorilla gorilla* X93347.1, *Bos taurus* AY526085.1, *Equus caballus* EF597513.1,Vi331.6 Neandertal AM948965 and *Cercopithicus albogularis monoides* (kindly provided by K. Finstermeier)) using MIA [18].

To determine fragment lengths of aligned reads, BWA [24] was used to align both fused sequence and remaining non-merged read pairs to the reference genomes. Samtools [25] was used to extract the inferred insert-size of aligned read-pairs. For aligned fused sequences, the length of the sequence was used. The statistical platform R [26] was used perform Spearman's correlation tests.

## Supporting Information

Table S1 List of samples with age, location and sequencing results, species used for long range PCR bait, museum ID, sample material and storage conditions.
(DOC)

Table S2 List of samples with sequencing analysis results pertaining to ancient DNA features. Listed are: deamination frequency of the 5′ end of fragments, the increase in purine frequency before the 5′ fragment ends, increase in adenine and guanine base pair frequencies before the 5′ ends, median lengths of the fragments, and basepairs per milligram (bp/mg) of sample material. All measurements, except bp/mg bone, were calculated for samples with at least 450 aligned reads. The others are denoted with a “NA” for not applicable.
(DOC)
