# Supplementary material for: Temporal Patterns of Nucleotide Misincorporations and DNA Fragmentation in Ancient DNA
Source: PLoS One. 2012 Mar 30;7(3):e34131. doi: 10.1371/journal.pone.0034131 (PMC3316601; doi:10.1371/journal.pone.0034131)
Supplement: Table S1 — List of samples with age, location and sequencing results, species used for long range PCR bait, museum ID, sample material and storage conditions. (DOC) [file pone.0034131.s001.doc]

**Sample Age Average ^a^ Aligned ^b^ Percent ^c^ LR-PCR Type of Condition of Number Animal (years) Location Coverage reads on target Bait Museum ID sample Storage**

| 4.56 | Monkey | 18 | Royal Museum for Central Africa (RMCA) | 203.3 | 49940 | 3.97 | Cercopithecus | 93.091-M-0039 | cartilage sample | Shot then stored since  then in museum |
| --- | --- | --- | --- | --- | --- | --- | --- | --- | --- | --- |
| 3.62 | Monkey | 18 | RMCA | 203.9 | 60691 | 6.32 | Cercopithecus | 93.091-M-0025 | cartilage sample | Shot then stored since  then in museum |
| 3.35 | Monkey | 24 | RMCA | 245.8 | 61019 | 27.81 | Cercopithecus | 87.060-M-0001 | cartilage sample | Shot then stored since  then in museum |
| 3.80 | Monkey | 26 | RMCA | 35.6 | 9216 | 14.17 | Cercopithecus | 86.025-M-0001 | cartilage sample | Shot then stored since  then in museum |
| 3.51 | Monkey | 26 | RMCA | 80.4 | 21159 | 27.11 | Cercopithecus | 86.025-M-0005 | cartilage sample | Shot then stored since  then in museum |
| 3.46 | Monkey | 41 | RMCA | 123.3 | 23301 | 3.50 | Cercopithecus | 77.048-M-0050 | dried skin sample | Shot then stored since  then in museum |
| 3.56 | Monkey | 41 | RMCA | 114.9 | 27413 | 2.85 | Cercopithecus | 73.029-M-0113 | dried skin sample | Shot then stored since  then in museum |
| 3.40 | Monkey | 42 | RMCA | 15.0 | 3260 | 1.34 | Cercopithecus | 35700 | dried skin sample | Shot then stored since  then in museum |
| 4.40 | Monkey | 46 | RMCA | 134.0 | 45631 | 2.22 | Cercopithecus | 33514 | dried skin sample | Shot then stored since  then in museum |
| 106 | Monkey | 48 | Smithsonian NMNH | 168.7 | 31334 | 52.30 | Human | 452514 | bone sample | Shot then stored since  then in museum,  bone sample taken before treatment |
| 3.43 | Monkey | 54 | RMCA | 96.2 | 19809 | 18.01 | Cercopithecus | 26170 | cartilage sample | Shot then stored since  then in museum |
| 4.64 | Monkey | 55 | RMCA | 231.1 | 56683 | 1.64 | Cercopithecus | 25441 | dried skin sample | Shot then stored since  then in museum |
| 3.70 | Monkey | 59 | RMCA | 88.1 | 19165 | 1.54 | Cercopithecus | 21115 | dried skin sample | Shot then stored since  then in museum |
| 4.27 | Monkey | 62 | RMCA | 4.4 | 883 | 1.40 | Cercopithecus | 19554 | bone sample | Shot then stored since  then in museum |
| 4.48 | Monkey | 71 | RMCA | 11.5 | 3027 | 0.30 | Cercopithecus | 17512 | dried skin sample | Shot then stored since  then in museum |
| 2.45 | Monkey | 74 | Museum für Naturkunde Berlin (MfN) | 63.2 | 14335 | 0.66 | Cercopithecus | 41171 | dried skin sample | Shot then stored since  then in museum |
| 4.19 | Monkey | 76 | RMCA | 26.6 | 6296 | 1.30 | Cercopithecus | 12839 | dried skin sample | Shot then stored since  then in museum |
| 2.29 | Monkey | 83 | MfN | 18.1 | 6513 | 0.82 | Cercopithecus | 40413 | cartilage sample | Shot then stored in museum,  treated with Leipzig cocktail |
| 107 | Gorilla | 83 | Powell-Cotton Museum | 1416.9 | 345619 | 21.60 | Gorilla | CAMI 83 | bone sample | Shot then stored since  then in museum |
| 3.59 | Monkey | 86 | RMCA | 665.6 | 144993 | 9.99 | Cercopithecus | 8559 | dried skin sample | Shot then stored since  then in museum |
| 2.49 | Monkey | 87 | MfN | 24.3 | 6661 | 0.32 | Cercopithecus | 40393 | cartilage sample | Shot then stored in museum,  treated with Leipzig cocktail |
| 3.16 | Monkey | 90 | RMCA | 47.2 | 13602 | 1.13 | Cercopithecus | 5780 | dried skin sample | Shot then stored since  then in museum |
| *3.54 | Monkey | 91 | RMCA | 0.5 | 169 | 1.63 | Cercopithecus | 5598 | bone sample | Shot then stored since  then in museum |
| 3.24 | Monkey | 98 | RMCA | 35.0 | 8447 | 1.45 | Cercopithecus | 1565 | dried skin sample | Shot then stored since  then in museum |
| 2.44 | Monkey | 98 | MfN | 32.8 | 7302 | 0.25 | Cercopithecus | 19079 | dried skin sample | Shot then stored since  then in museum |
| *2.22 | Monkey | 100 | MfN | 0.1 | 48 | 0.00 | Cercopithecus | 15833 | bone sample | Shot then stored since  then in museum |
| 2.33 | Monkey | 100 | MfN | 30.4 | 8466 | 0.75 | Cercopithecus | 15776 | bone sample | Shot then stored since  then in museum |
| 113 | Gorilla | 100 | MfN | 449.70 | 115924 | 30 | Gorilla | 83553 | bone sample | Shot then stored in  museum,  Ponal (glue) treated |
| 111 | Gorilla | 100 | MfN | 12.60 | 3680 | 3.1 | Gorilla | 12792 | tissue sample | Shot then stored in  museum,  Ponal (glue) treated |
| 110 | Gorilla | 100 | MfN | 125.10 | 41737 | 16.5 | Gorilla | 12790 | cartilage sample | Shot then stored in  museum,  Ponal (glue) treated |
| 109 | Gorilla | 100 | MfN | 940.40 | 227022 | 40.5 | Gorilla | 12795 | bone sample | Shot then stored in  museum,  Ponal (glue) treated |
| 2.27 | Monkey | 103 | MfN | 92.1 | 35432 | 6.08 | Cercopithecus | 13690 | bone sample | Shot then stored in  museum,  treated with Leipzig cocktail |
| 117 | Gorilla | 103 | MfN | 41.30 | 11421 | 4.8 | Gorilla | 83549 | bone sample | Shot then stored in  museum,  Ponal (glue) treated |
| 116 | Gorilla | 103 | MfN | 133.50 | 38667 | 15.4 | Gorilla | 83541 | bone sample | Shot then stored in  museum,  Ponal (glue) treated |
| 115 | Gorilla | 103 | MfN | 26.30 | 7006 | 9.3 | Gorilla | 83542 | skin sample | Shot then stored in  museum,  Ponal (glue) treated |
| 114 | Gorilla | 103 | MfN | 27.70 | 7849 | 2.9 | Gorilla | 83570 | cartilage sample | Shot then stored in  museum,  Ponal (glue) treated |
| *2.6 | Monkey | 104 | MfN | 0.1 | 20 | 0.00 | Cercopithecus | 87673 | bone sample | Shot then stored since  then in museum |
| 112 | Gorilla | 104 | MfN | 156.00 | 43093 | 8.6 | Gorilla | 48170 | bone sample | Shot then stored in  museum,  Ponal (glue) treated |
| 4.72 | Monkey | 106 | RMCA | 5.6 | 1603 | 2.68 | Cercopithecus | 92-124-M-0003 | cartilage sample | Shot then stored in museum,  treated with Leipzig cocktail |
| *2.32 | Monkey | 109 | MfN | 0.3 | 99 | 0.02 | Cercopithecus | 12546 | bone sample | Shot then stored since  then in museum |
| 2.42 | Monkey | 115 | MfN | 3.3 | 911 | 0.07 | Cercopithecus | 9753 | tissue sample | Shot then stored since  then in museum |
| 2.46 | Monkey | 115 | Berlin NK Museum | 74.1 | 14240 | 1.34 | Cercopithecus | 8560 | bone sample | Shot then stored in museum,  treated with Leipzig cocktail |
| 2.41 | Monkey | 117 | MfN | 23.2 | 7170 | 0.60 | Cercopithecus | 7162 | bone sample | Shot then stored since  then in museum |
| *2.4 | Monkey | 117 | MfN | 0.1 | 23 | 0.00 | Cercopithecus | 33223 | bone sample | Shot then stored since  then in museum |
| 53 | Horse | 500 - 600 | Sittard, Holland | 3.321 | 825 | 0.2 | Horse | 16695-1956 | bone sample | Excavated in 2006,  stored since then in cardboard boxes |
| 54 | Horse | 500 - 600 | Sittard, Holland | 49.38 | 13032 | 5.0 | Horse | 16695-1942 | bone sample | Excavated in 2006,  stored since then in cardboard boxes |
| 55 | Horse | 500 - 600 | Sittard, Holland | 2.52 | 722 | 0.3 | Horse | 16695-1955 | bone sample | Excavated in 2006,  stored since then in cardboard boxes |
| 56 | Horse | 500 - 600 | Sittard, Holland | 2.962 | 776 | 0.3 | Horse | 16695-1954 | bone sample | Excavated in 2006,  stored since then in cardboard boxes |
| 57 | Horse | 500 - 600 | Sittard, Holland | 1.929 | 537 | 0.1 | Horse | 16695-1951 | bone sample | Excavated in 2006,  stored since then in cardboard boxes |
| *128 | Horse | 600 - 700 | Sittard, Holland | 1.6 | 261 | 0.0 | Horse | 27134-378 | bone sample | Excavated in 2006,  stored since then in cardboard boxes |
| *129 | Horse | 600 - 700 | Sittard, Holland | 0.3 | 105 | 0.0 | Horse | 27134-379 | bone sample | Excavated in 2006,  stored since then in cardboard boxes |
| *130 | Horse | 600 - 700 | Sittard, Holland | 0.4 | 72 | 0.0 | Horse | 27134-379 | bone sample | Excavated in 2006,  stored since then in cardboard boxes |
| *131 | Horse | 600 - 700 | Sittard, Holland | 0.3 | 76 | 0.0 | Horse | 27134-381 | bone sample | Excavated in 2006,  stored since then in cardboard boxes |
| *52 | Horse | 600 - 700 | Sittard, Holland | 0.1 | 31 | 0.0 | Horse | 27134-323 | bone sample | Excavated in 2006,  stored since then in cardboard boxes |
| 77 | Cow | 800 - 900 | Halle, Germany | 12.21 | 2888 | 0.7 | Cow | 2005: 22 708b | bone sample | Excavated in 1994,  stored since then in  plastic bags |
| 78 | Cow | 800 - 900 | Halle, Germany | 324 | 84623 | 5.0 | Cow | 2005: 22 708b | bone sample | Excavated in 1994,  stored since then in  plastic bags |
| 79 | Cow | 800 - 900 | Halle, Germany | 8.5 | 1952 | 3.5 | Cow | 2005: 22 708b | bone sample | Excavated in 1994,  stored since then in  plastic bags |
| 80 | Cow | 800 - 900 | Halle, Germany | 913.3 | 218031 | 13.5 | Cow | 2005: 22 708b | bone sample | Excavated in 1994,  stored since then in  plastic bags |
| 82 | Cow | 800 - 900 | Halle, Germany | 100.4 | 24739 | 4.0 | Cow | 2005: 22 708b | bone sample | Excavated in 1994,  stored since then in  plastic bags |
| 28 | Horse | 1200 - 1300 | Loebnitz, Germany | 7.0 | 1565 | 2.4 | Horse | 66: 386 | bone sample | Excavated in 1966,  stored since then in cardboard boxes |
| 35 | Horse | 1200 - 1300 | Loebnitz, Germany | 3.6 | 802 | 15.1 | Horse | 66: 387 | bone sample | Excavated in 1966,  stored since then in cardboard boxes |
| *1 | Horse | 1200 - 1300 | Loebnitz, Germany | 9.1 | 379 | 0.4 | Horse | 66: 384 | bone sample | Excavated in 1966,  stored since then in cardboard boxes |
| *4 | Horse | 1200 - 1300 | Loebnitz, Germany | 5.2 | 157 | 13.6 | Horse | 66: 385 | bone sample | Excavated in 1966,  stored since then in cardboard boxes |
| *41 | Horse | 1200 - 1300 | Loebnitz, Germany | 6.2 | 415 | 0.1 | Horse | 67: 281 | bone sample | Excavated in 1967,  stored since then in cardboard boxes |
| 71 | Horse | 1400 - 1600 | Deersheim, Germany | 3.5 | 793 | 0.1 | Horse | 72: 400II | bone sample | Excavated in 1972,  stored since then in cardboard boxes |
| *44 | Horse | 1400 - 1600 | Deersheim, Germany | 2.0 | 237 | 0.3 | Horse | 70: 201/203 | bone sample | Excavated in 1963/64,  stored since then in cardboard boxes |
| *45 | Horse | 1400 - 1600 | Deersheim, Germany | 1.2 | 78 | 0.2 | Horse | 70: 207a | bone sample | Excavated in 1963/64,  stored since then in cardboard boxes |
| *62 | Horse | 1400 - 1600 | Deersheim, Germany | 0.4 | 26 | 0.1 | Horse | 70: 208 | bone sample | Excavated in 1963/64,  stored since then in cardboard boxes |
| *64 | Horse | 1400 - 1600 | Deersheim, Germany | 2.1 | 215 | 0.1 | Horse | 70: 209 | bone sample | Excavated in 1963/64,  stored since then in cardboard boxes |
| *86 | Horse | 1400 - 1600 | Deersheim, Germany | 4.1 | 254 | 0.3 | Horse | 72: 401 | bone sample | Excavated in 1972,  stored since then in cardboard boxes |
| *123 | Horse | 1730 - 1900 | Sittard, Holland | 1.5 | 384 | 0.1 | Horse | 16695-1761 | bone sample | Excavated in 2008,  stored since then in cardboard boxes |
| 124 | Horse | 1730 - 1900 | Sittard, Holland | 8.7 | 2492 | 0.7 | Horse | 16695-1730 | bone sample | Excavated in 2008,  stored since then in cardboard boxes |
| *125 | Horse | 1730 - 1900 | Sittard, Holland | 0.8 | 173 | 0.0 | Horse | 16695-1719 | bone sample | Excavated in 2008,  stored since then in cardboard boxes |
| 126 | Horse | 1730 - 1900 | Sittard, Holland | 3.4 | 849 | 1.4 | Horse | 16695-1728 | bone sample | Excavated in 2008,  stored since then in cardboard boxes |
| 127 | Horse | 1730 - 1900 | Sittard, Holland | 27.9 | 7153 | 2.4 | Horse | 16695-1729 | bone sample | Excavated in 2008,  stored since then in cardboard boxes |
| 118 | Horse | 1900 - 2400 | Sittard, Holland | 14.0 | 3619 | 4.1 | Horse | 27134-777 | bone sample | Excavated in 2008,  stored since then in cardboard boxes |
| *119 | Horse | 1900 - 2400 | Sittard, Holland | 2.9 | 382 | 0.1 | Horse | 27134-768 | bone sample | Excavated in 2008,  stored since then in cardboard boxes |
| 120 | Horse | 1900 - 2400 | Sittard, Holland | 32.8 | 8233 | 2.8 | Horse | 27134-778 | bone sample | Excavated in 2008,  stored since then in cardboard boxes |
| 121 | Horse | 1900 - 2400 | Sittard, Holland | 68.7 | 15563 | 17.3 | Horse | 27134-1294 | bone sample | Excavated in 2008,  stored since then in cardboard boxes |
| 122 | Horse | 1900 - 2400 | Sittard, Holland | 34.1 | 8473 | 1.6 | Horse | 27134-797 | bone sample | Excavated in 2008,  stored since then in cardboard boxes |

**^a^** The average number of reads that cover any given position of the mitochondrial genome (duplicate fragments were removed)

**^b^** The number of fragments that aligned to the mitochondrial genome after duplicate fragments were removed

**^c^** The percent of all fragments (including duplicates) that aligned to the mitochondrial genome

***** These samples were used only in a subset of analyses, because the number of aligned reads was too low
