# Supplementary material for: Temporal Patterns of Nucleotide Misincorporations and DNA Fragmentation in Ancient DNA
Source: PLoS One. 2012 Mar 30;7(3):e34131. doi: 10.1371/journal.pone.0034131 (PMC3316601; doi:10.1371/journal.pone.0034131)
Supplement: Table S2 — List of samples with sequencing analysis results pertaining to ancient DNA features. Listed are: deamination frequency of the 5′ end of fragments, the increase in purine frequency before the 5′ fragment ends, increase in adenine and guanine base pair frequencies before the 5′ ends, median lengths of the fragments, and basepairs per milligram (bp/mg) of sample material. All measurements, except bp/mg bone, were calculated for samples with at least 450 aligned reads. The others are denoted with a “NA” for not applicable. (DOC) [file pone.0034131.s002.doc]

**Freq of Increase in freq Increase in freq Increase in freq**

**Sample Age deamination of purines of adenine of guanine Median bp/mg**

**Number Animal (years) at 5’ end before 5’ end before 5’ before 5’ length material**

| 4.56 | Monkey | 18 | 0.004 | 0.272 | 0.169 | 0.103 | 60 | 123398 |
| --- | --- | --- | --- | --- | --- | --- | --- | --- |
| 3.62 | Monkey | 18 | 0.011 | 0.221 | 0.122 | 0.098 | 54 | 125105 |
| 3.35 | Monkey | 24 | 0.009 | 0.377 | 0.279 | 0.098 | 70 | 152533 |
| 3.8 | Monkey | 26 | 0.011 | 0.35 | 0.229 | 0.121 | 51 | 21820 |
| 3.51 | Monkey | 26 | 0.008 | 0.364 | 0.244 | 0.119 | 59 | 41498 |
| 3.46 | Monkey | 41 | 0.02 | 0.418 | 0.285 | 0.133 | 81 | 53628 |
| 3.56 | Monkey | 41 | 0.017 | 0.411 | 0.299 | 0.112 | 165 | 50750 |
| 3.4 | Monkey | 42 | 0.034 | 0.326 | 0.211 | 0.115 | 55 | 6739 |
| 4.4 | Monkey | 46 | 0.011 | 0.303 | 0.2 | 0.103 | 51 | 52110 |
| 106 | Monkey | 48 | 0.019 | 0.156 | 0.066 | 0.09 | 124 | 1941450 |
| 3.43 | Monkey | 54 | 0.021 | 0.367 | 0.231 | 0.137 | 81 | 37950 |
| 4.64 | Monkey | 55 | 0.006 | 0.41 | 0.276 | 0.134 | 61 | 120431 |
| 3.7 | Monkey | 59 | 0.019 | 0.408 | 0.263 | 0.145 | 62 | 32665 |
| 4.27 | Monkey | 62 | 0.03 | 0.4 | 0.263 | 0.137 | 65 | 1418 |
| 4.48 | Monkey | 71 | 0.03 | 0.405 | 0.271 | 0.134 | 52 | 10556 |
| 2.45 | Monkey | 74 | 0.03 | 0.267 | 0.157 | 0.11 | 61 | 122443 |
| 4.19 | Monkey | 76 | 0.03 | 0.432 | 0.295 | 0.138 | 55 | 7140 |
| 2.29 | Monkey | 83 | 0.038 | 0.414 | 0.299 | 0.115 | 46 | 4734 |
| 107 | Gorilla | 83 | 0.039 | 0.295 | 0.185 | 0.11 | 88 | 48889 |
| 3.59 | Monkey | 86 | 0.019 | 0.41 | 0.257 | 0.154 | 77 | 251783 |
| 2.49 | Monkey | 87 | 0.038 | 0.33 | 0.214 | 0.115 | 55 | 7899 |
| 3.16 | Monkey | 90 | 0.036 | 0.414 | 0.3 | 0.114 | 50 | 17500 |
| 3.54 | Monkey | 91 | NA | NA | NA | NA | NA | 183 |
| 3.24 | Monkey | 98 | 0.028 | 0.429 | 0.29 | 0.139 | 170 | 10785 |
| 2.44 | Monkey | 98 | 0.05 | 0.357 | 0.22 | 0.136 | 62 | 20778 |
| 2.33 | Monkey | 100 | 0.04 | 0.368 | 0.253 | 0.114 | 54 | 478 |
| 2.22 | Monkey | 100 | NA | NA | NA | NA | NA | 147519 |
| 113 | Gorilla | 100 | 0.057 | 0.112 | 0.06 | 0.052 | 75 | 27283 |
| 111 | Gorilla | 100 | 0.157 | 0.119 | 0.085 | 0.034 | 54 | 28997 |
| 110 | Gorilla | 100 | 0.06 | 0.252 | 0.203 | 0.049 | 53 | 84809 |
| 109 | Gorilla | 100 | 0.026 | 0.176 | 0.118 | 0.057 | 85 | 209015 |
| 117 | Gorilla | 103 | 0.092 | 0.167 | 0.114 | 0.054 | 58 | 14506 |
| 116 | Gorilla | 103 | 0.059 | 0.119 | 0.035 | 0.084 | 59 | 168698 |
| 115 | Gorilla | 103 | 0.164 | 0.142 | 0.084 | 0.058 | 60 | 1192643 |
| 114 | Gorilla | 103 | 0.098 | 0.136 | 0.08 | 0.056 | 56 | 1552958 |
| 2.27 | Monkey | 103 | 0.025 | 0.318 | 0.229 | 0.089 | 44 | 133704 |
| 112 | Gorilla | 104 | 0.127 | 0.123 | 0.036 | 0.087 | 64 | 3716 |
| 2.6 | Monkey | 104 | NA | NA | NA | NA | NA | 25478 |
| 4.72 | Monkey | 106 | 0.02 | 0.372 | 0.309 | 0.063 | 50 | 2785 |
| 2.32 | Monkey | 109 | NA | NA | NA | NA | NA | 598 |
| 2.42 | Monkey | 115 | 0.049 | 0.301 | 0.16 | 0.141 | 53 | 6684 |
| 2.46 | Monkey | 115 | 0.041 | 0.368 | 0.259 | 0.108 | 73 | 50016 |
| 2.41 | Monkey | 117 | 0.03 | 0.29 | 0.178 | 0.111 | 52 | 1190 |
| 2.4 | Monkey | 117 | NA | NA | NA | NA | NA | 29101 |
| 53 | Horse | 500 - 600 | 0.118 | 0.122 | 0.023 | 0.099 | 61 | 3574 |
| 54 | Horse | 500 - 600 | 0.18 | 0.132 | 0.055 | 0.077 | 64 | 3966 |
| 55 | Horse | 500 - 600 | 0.192 | 0.105 | 0.047 | 0.058 | 56 | 5630 |
| 56 | Horse | 500 - 600 | 0.214 | 0.156 | 0.072 | 0.084 | 62 | 32790 |
| 57 | Horse | 500 - 600 | 0.127 | 0.172 | 0.064 | 0.108 | 64 | 233172 |
| 128 | Horse | 600-700 | NA | NA | NA | NA | NA | 107 |
| 129 | Horse | 600-700 | NA | NA | NA | NA | NA | 33 |
| 130 | Horse | 600-700 | NA | NA | NA | NA | NA | 23 |
| 131 | Horse | 600-700 | NA | NA | NA | NA | NA | 27 |
| 52 | Horse | 600-700 | NA | NA | NA | NA | NA | 12 |
| 77 | Cow | 800 - 900 | 0.215 | 0.12 | 0.081 | 0.04 | 66 | 252 |
| 78 | Cow | 800 - 900 | 0.253 | 0.212 | 0.128 | 0.084 | 68 | 362 |
| 79 | Cow | 800 - 900 | 0.273 | 0.151 | 0.094 | 0.057 | 76 | 415 |
| 80 | Cow | 800 - 900 | 0.27 | 0.175 | 0.1 | 0.076 | 66 | 716 |
| 82 | Cow | 800 - 900 | 0.146 | 0.086 | 0.035 | 0.051 | 68 | 7350 |
| 28 | Horse | 1200 - 1300 | 0.236 | 0.179 | 0.039 | 0.14 | 73 | 4288 |
| 35 | Horse | 1200 - 1300 | 0.17 | 0.121 | 0.051 | 0.069 | 75 | 74072 |
| 1 | Horse | 1200 - 1300 | NA | NA | NA | NA | NA | 1563 |
| 4 | Horse | 1200 - 1300 | NA | NA | NA | NA | NA | 864 |
| 41 | Horse | 1200 - 1300 | NA | NA | NA | NA | NA | 2080 |
| 71 | Horse | 1400 - 1600 | 0.379 | 0.307 | 0.061 | 0.246 | 68 | 2854 |
| 44 | Horse | 1400 - 1600 | NA | NA | NA | NA | NA | 1132 |
| 45 | Horse | 1400 - 1600 | NA | NA | NA | NA | NA | 178 |
| 62 | Horse | 1400 - 1600 | NA | NA | NA | NA | NA | 115 |
| 64 | Horse | 1400 - 1600 | NA | NA | NA | NA | NA | 842 |
| 86 | Horse | 1400 - 1600 | NA | NA | NA | NA | NA | 753 |
| 124 | Horse | 1730 - 1900 | 0.162 | 0.206 | 0.1 | 0.106 | 62 | 499 |
| 126 | Horse | 1730 - 1900 | 0.132 | 0.137 | 0.07 | 0.067 | 56 | 1334 |
| 127 | Horse | 1730 - 1900 | 0.137 | 0.196 | 0.082 | 0.114 | 62 | 4013 |
| 123 | Horse | 1730 - 1900 | NA | NA | NA | NA | NA | 184 |
| 125 | Horse | 1730 - 1900 | NA | NA | NA | NA | NA | 61 |
| 119 | Horse | 1900 - 2400 | NA | NA | NA | NA | NA | 136 |
| 118 | Horse | 1900 - 2400 | 0.123 | 0.135 | 0.047 | 0.089 | 61 | 1941 |
| 120 | Horse | 1900 - 2400 | 0.11 | 0.153 | 0.063 | 0.09 | 64 | 4311 |
| 121 | Horse | 1900 - 2400 | 0.162 | 0.184 | 0.102 | 0.082 | 66 | 4689 |
| 122 | Horse | 1900 - 2400 | 0.129 | 0.18 | 0.073 | 0.107 | 76 | 10165 |
